# Anthocyanin Extraction from Jaboticaba Skin (*Myrciaria cauliflora* Berg.) Using Conventional and Non-Conventional Methods

**DOI:** 10.3390/foods11060885

**Published:** 2022-03-20

**Authors:** Gabriela Nunes Mattos, Manuela Cristina Pessanha de Araújo Santiago, Ana Carolina Sampaio Doria Chaves, Amauri Rosenthal, Renata Valeriano Tonon, Lourdes Maria Correa Cabral

**Affiliations:** 1 Programa de Pós-Graduação em Ciência de Alimentos, Instituto de Química, Universidade Federal do Rio de Janeiro, Rio de Janeiro 21941-909, RJ, Brazil; nunesmattos.gabriela@gmail.com; 2 Embrapa Agroindústria de Alimentos, Av. das Américas, 29501, Rio de Janeiro 23020-470, RJ, Brazil; manuela.santiago@embrapa.br (M.C.P.d.A.S.); ana.chaves@embrapa.br (A.C.S.D.C.); amauri.rosenthal@embrapa.br (A.R.); renata.tonon@embrapa.br (R.V.T.)

**Keywords:** jaboticaba skin, by-product, ultrasound-assisted extraction, high hydrostatic pressure-assisted extraction, anthocyanins, bioactive compounds

## Abstract

This study evaluated the effect of different extraction technologies and conditions in order to obtain jaboticaba skin extracts. Firstly, the skins were extracted by conventional extraction, according to a rotatable central composite design, varying ethanol concentration, solid:liquid ratio, and temperature. Next, ultrasound-assisted extraction was performed using different power densities and times. Finally, high-pressure extractions were performed with varying pressures and times. For agitated bed extraction, the highest anthocyanin content was observed for ethanol concentrations varying between 60% and 80%. Thus, the independent variables which more influenced anthocyanin content were ethanol concentration and solid:liquid ratio. Folin–Ciocalteu reducing capacity was linearly affected by the increase in temperature. Ethanol concentration was the variable that most influenced ABTS+. On the other hand, the increase in ethanol concentration decreased the antioxidant capacity by ABTS+. Considering the ultrasound extraction, increasing its power did not affect total monomeric anthocyanins content, while the increase in process time had better yields. The highest antioxidant capacity and total monomeric anthocyanins were found for the highest extraction time. Similarly, with ultrasound, the increase in high hydrostatic-assisted extraction time positively influenced anthocyanin content and antioxidant capacity. As a result, the ultrasound-assisted method was found to be the best extraction technology for anthocyanins recovery.

## 1. Introduction

Brazilian fruit crops have great world relevance, as Brazil is considered one of the most important fruit producers in the world, cultivating over 2 million of hectares around the country. Nowadays, Brazil is the third largest producer, just below China and India [1].

Jaboticaba (*Myrciaria cauliflora* Berg.) is a native Brazilian crop, spread across the country, being mainly consumed in natura, and it possesses good sensory characteristics. However, processing represents a good alternative to increase its yield and shelf life, in the form of juices, jellies, fermented drinks, vinegar, and others [2,3,4,5]. Some researchers state that jaboticaba is rich in vitamins and minerals such as vitamin C, potassium, phosphor, iron, calcium, in addition to having a high content of bioactive compounds, especially phenolic compounds, such as anthocyanins and tannins [4,6]. The fruit has gained notoriety since studies have shown its functional properties, such as antioxidant capacity, anti-inflammatory activity, and other health benefits [7,8,9,10,11].

Jaboticaba processing results in the generation of large amounts of waste, also known as by-products, basically consisting of peels and seeds. The peels are responsible for 50% of the fruit weight, with a high content of cyanidin-3-glycoside as the major anthocyanin [3]. Traditionally, this by-product is thrown away. However, the use of this residue as an anthocyanin source for functional ingredients can be a promising alternative to decrease environmental impact and add value to the crop [12].

There are many techniques to extract bioactive compounds from vegetal matrices. Conventional extraction by mechanical agitation is in general a long process that can use high temperatures, which can cause undesirable hydrolysis and/or oxidation of these compounds. In this sense, several emerging (or non-conventional) methods have been studied lately, aiming to reduce the processing time and improve the extraction efficiency. Among them, some emerging technologies have been gaining attention, such as ultrasound and high hydrostatic pressure.

Ultrasound-assisted extraction (UAE) is an excellent alternative that involves the generation of cavitation flows, which generates adiabatic compression of gases inside the bubbles, resulting in bubble collapse, affecting the cell membrane structure, and improving the extraction of bioactive compounds [13]. The interest on ultrasound-assisted methods is continuously increasing, due to its low cost, reduced processing time, simple operation, high extraction efficiency, and good reproducibility [14]. Rodrigues [15] studied the influence of UAE conditions on the recovery of phenolic compounds from jaboticaba skin using different pH (2.0 to 5.0) and ethanolic extract solutions (3.14% to 46.86%). The authors found that 46% ethanolic solution, pH 3.4, and 60 min of extraction were the best conditions for monomeric anthocyanins extraction. Despite the authors using HCl in order to obtain better results of extraction, this experiment avoids usage of HCL in order to obtain green extract that could be used as a food ingredient.

High hydrostatic pressure-assisted extraction (HHE) is another important processing technology that uses warm temperatures, besides inactivating microorganisms, enzymes retain the nutrients [16]. During the process, several structural changes occur due to the application of pressures in the range of 100 to 800 MPa, which results in an increase in solvent permeability, as well as better extraction yields and shorter process times [17,18,19,20,21]. To the best of our knowledge, there is no report of studies focused on HHE of phenolic compounds from jaboticaba skins. Pimenta Inada et al. [22] evaluated the effect of different methods on the phenolic compounds on jaboticaba peel and seed using high hydrostatic pressure as a pretreatment to increase phenolic compound extractability. The authors observed that high hydrostatic pressure was ineffective in improving bioactive compound extractability on jaboticaba skins and seeds. Cascaes Teles et al. [23] studied HHE extraction of grape pomace with presence and absence of enzymes and observed that high hydrostatic pressure promoted good extraction of bioactive compounds, improving enzyme activity.

The use of jaboticaba skin as a raw material to obtain high added-value ingredients for food and pharmaceutical industry is a good technological alternative that could be better explored which also would result in a positive environmental impact. Therefore, the aim of this study was to obtain an anthocyanin-rich extract from jaboticaba skin using a conventional (agitated bed extraction) and two non-conventional (UAE and HHE) technologies. Firstly, the effect of process conditions (ethanol concentration, temperature, and solid:liquid ratio) on the anthocyanin extraction was evaluated for the conventional method. Then, in selected conditions, the non-conventional methods were evaluated for different processing times and conditions.

## 2. Materials and Methods

### 2.1. Materials

Jaboticaba (*Myrciaria cauliflora* Berg.) was purchased in a local market (Rio de Janeiro, RJ, Brazil).

For extractions, ethanol (Vetec, Rio de Janeiro, Brazil) and distilled water were used. For analysis of phenolic compounds and antioxidant capacity, Folin–Ciocalteau reagent, ABTS+, K2SO5, and Trolox were purchased from Sigma-Aldrich (St. Louis, MO, USA). We also acquired sodium bicarbonate (Alphatec, Macaé, RJ, Brazil), formic acid (Merck, Rahway, NJ, USA), and methanol (Tedia, Fairfield, CA, USA). Standard of the 3-O-glucoside of cyanidin was isolated from natural source, with purities greater than 99%.

### 2.2. Preparation of Jaboticaba Pomace

The skins were obtained after pulping the fruits in a pulping machine (Bonina 0.25 dF, Itabuna, BA, Brazil). The jaboticaba skin was dried at 60 °C for 24 h in a convective dryer, milled in a LM 3600 hammer grinding mill (Perten Instruments AB, Hägersten, Sweden), and stored at 18 °C in the dark until use.

### 2.3. Agitated Bed Extraction

The agitated bed extraction was performed in an incubator with orbital shaking 430/RDBP (Nova Ética, Vargem Grande Paulista, SP, Brazil) at 150 rpm for one hour, using a hydroethanolic solution. After extraction, this mixture was filtered using a Whatman No. 1 filter paper. The extract was stored at −18 °C.

The experiment was carried out according to a rotatable central composite design, based on response surface methodology, considering three independent variables: ethanol concentration (10–90% *v*/*v*), solid:liquid ratio (1:5–1:13) and temperature (23–57 °C). Five levels were chosen to evaluate the combinations, totaling 17 runs (Table 1).

The following polynomial equation was fitted to the data:

(1) $$y=\beta_{0}+\beta_{1}x_{1}+\beta_{2}x_{2}+\beta_{3}x_{3}+\beta_{11}x_{1}^{2}+\beta_{22}x_{2}^{2}+\beta_{33}x_{3}^{2}+\beta_{12}x_{1}x_{2}+\beta_{13}x_{1}x_{3}+\beta_{23}x_{2}x_{3}$$

where, *B_n_* is the constant regression coefficients; *y* is the response (total phenolic content or antioxidant capacity); and *x*_1_, *x*_2_, and *x*_3_ are the coded independent variables (temperature, ethanol concentration, and solid:liquid ratio, respectively).

The results were analyzed by response surface methodology and Pareto chart. The ANOVA and test for the lack of fit were carried out using the Statistica 7.0 software (StatSoft, Tulsa, OK, USA).

The best extraction conditions were selected based on the anthocyanin content and antioxidant capacity. The ethanol concentration and solid:liquid ratio selected as the most adequate conditions were used in the extraction by non-conventional methods (UAE and HHE).

### 2.4. Ultrasound-Assisted Extraction (UAE)

The UAE was performed using an ultrasonic system UIP1000hdT (Hielscher Ultrasonics, Teltow, Germany) with a sonotrode (BS4d34), 34 mm in diameter, and a sensor for the sample temperature control, using a frequency of 20 kHz. The jaboticaba skins were mixed with hydroethanolic solution with a solid:liquid ratio of 1:13 in a beaker of 250 mL. In order to avoid excessive heating, the beaker was placed into an ice bath. Extractions were performed using three powers densities (150, 250, and 350 W/L) and three times (1, 3, and 10 min). The sonotrode was submerged in the solution. Extractions were performed in triplicate. The mixtures were vacuum-filtered and stored at −18 °C.

### 2.5. High Hydrostatic Pressure-Assisted Extraction (HHE)

For HHE, the mixture (jaboticaba skins and hydroethanolic solution, in a solid:liquid ratio of 1:13) was packed in polyethylene bags (Selovac 200 B II, Selovac, São Paulo, Brazil). HHE was conducted in a Stansted Fluid Power pressurizer (Model S-FL-850-9-W; Stansted Fluid Power Ltd., Harlow, United Kingdom). Extractions were performed using three levels of pressure (200, 300, and 400 MPa) and three times (5, 10, and 15 min). Extractions were performed in triplicate. The mixtures were vacuum-filtered and stored at −18 °C.

### 2.6. Analytical Methods

#### 2.6.1. Total Monomeric Anthocyanin Content

The anthocyanin content was determined as described by de Brito et al. [24] and adapted by Santiago et al. [25]. For anthocyanin determination, extracts were filtered through a hydrophilic membrane (0.45 μm) (Millipore, Bedford, MA, USA), transferred to a vial, injected into the chromatograph, and analyzed in a Waters Alliance High Performance Liquid Chromatograph model 2690/5 (Waters, Milford, MA, USA) coupled to a Waters 2996 photodiode array detector. Chromatographic separation of compounds was conducted using a reverse column (C18, Thermo BDS Hypersil, 100 × 4.6 mm, 2.4 μm). The mobile phase consisted of 5% formic acid aqueous solution and acetonitrile with a flow rate of 1.0 mL·min^−1^ and injection volume of 20 μL. The gradient for solvent B was: 5% in 0 min, 7% in 2 min, 10% in 10 min, 13% in 15 min, 15% in 16 min, 17% in 20 min, 20% in 30 min, and 5% in 33 min.

The anthocyanin content was expressed as mg of cyanidin-3-O-glucoside equivalent per 100 g of dry weight (mg c3g. 100 g^−1^ dw). The results were obtained at 520 nm.

#### 2.6.2. Antioxidant Capacity

##### Folin–Ciocalteu Reducing Capacity

The antioxidant capacity by Folin–Ciocalteu reducing capacity was measured according to the methodology described by Singleton and Rossi [26] and George et al. [27]. Briefly, a sample of 250 μL was mixed with 1.25 mL of Folin–Ciocalteu reagent and 1 mL of sodium bicarbonate 7.5%. After two minutes, the mixture was incubated (50 °C) for 15 min. After this, the tubes were cooled, and the absorbance was read at 760 nm. The results were expressed as mg gallic acid equivalent (GAE) per gram of dry weight.

##### ABTS+ Cationic Radical Scavenging Activity

The ABTS+ antioxidant capacity was measured according to Re et al. [28]. For the ABTS+ preparation, 5 mL of the 7 mM ABTS+ aqueous solution was added to 88 μL of 140 mM potassium persulfate solution, kept in a closed flask and left to stand in the dark for at least 14 h. The ABTS+ stock solution was diluted in ethanol 95%. For the reaction, 3 mL of ABTS+ ethanolic solution was added to 30 μL of extract and after 6 min, the absorbance was read at 734 nm. Results were expressed as μmol Trolox equivalent per gram of dry weight.

### 2.7. Statistical Analysis

Data were evaluated by analysis of variance (ANOVA) followed by Tukey’s comparison using Statistica 7.0 (StatSoft, Tulsa, OK, USA), where the statistically significantly difference was *p* < 0.05. Graphs were generated using GraphPad 5.04 (GraphPad Software, San Diego, CA, USA). The results were expressed as mean ± standard deviation.

## 3. Results

### 3.1. Agitated Bed Extraction

Results obtained in agitated bed extraction for total monomeric anthocyanins content and antioxidant capacity by ABTS+ and Folin–Ciocalteu reducing capacity assays are shown in Table 1 and Table 2.

Anthocyanin content varied from 58.92 to 284.09 mg cyanidin-3-glucoside 100 g^−1^ of jaboticaba skin. These results are higher than those found by Silva et al. [29]. in the jaboticaba peel extract obtained by conventional extraction using 70% ethanolic solution during 48 h (48.06 mg of anthocyanins 100 g^−1^). On the other hand, these values were lower than those reported by Albuquerque et al. [30] who studied jaboticaba peel extraction using acidified ethanolic solution ethanol: water (80% *v*/*v*) and 0.1% citric acid for 1 h (2454 mg of cyanidin-3-glucoside 100 g^−1^ of skin dry weight).

**Figure 1:**
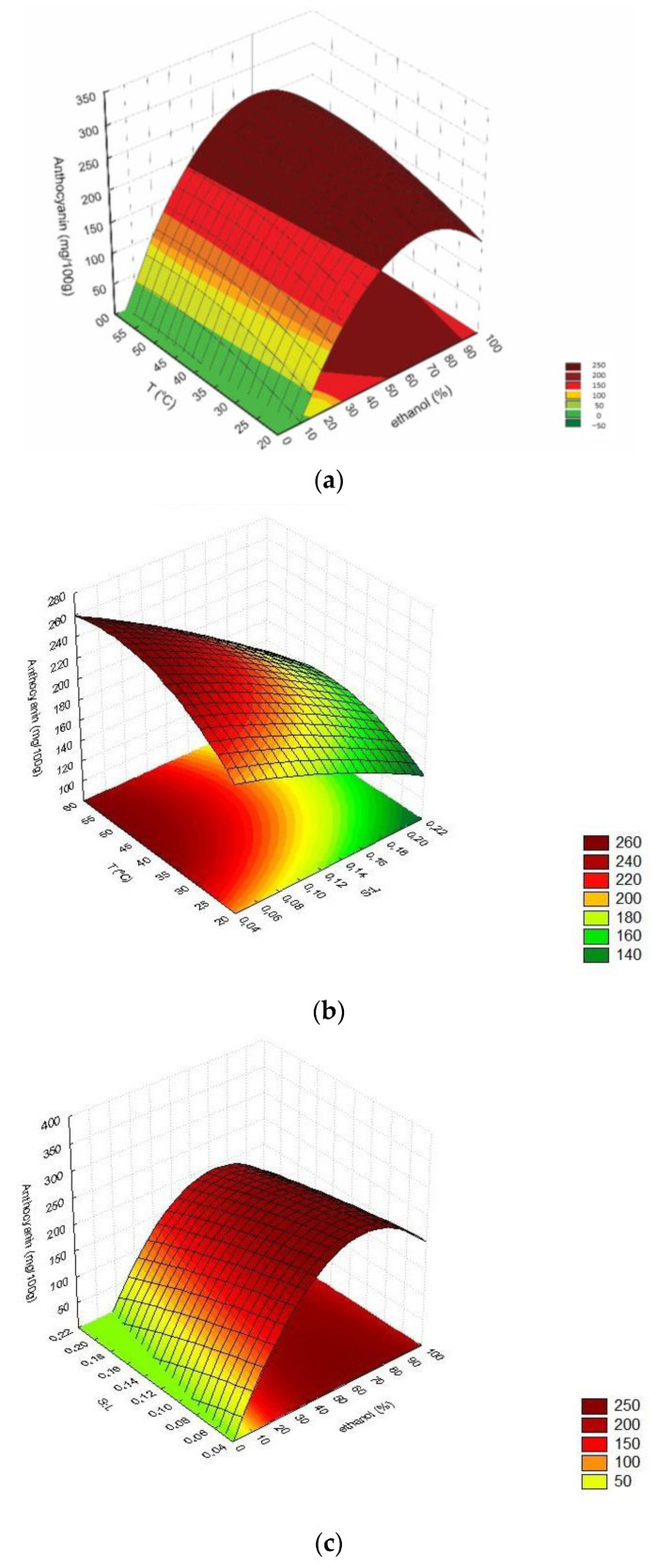
Response surfaces for total monomeric anthocyanins content: (**a**) Ethanol (%) × Temperature (°C) for S:L of 1:10; (**b**) S:L ratio × Temperature (°C) for ethanol concentration of 50%; (**c**) S:L ratio × Ethanol (%) for a temperature of 40 °C.

All the evaluated process conditions significantly affected anthocyanin extraction (*p* < 0.05). According to Figure 1, higher ethanol concentrations resulted in better extraction yields, as previously reported in the literature for cherries and purple yam [31,32]. The highest anthocyanin content was observed for ethanol concentrations varying between 60% and 80%. In contrast, lower or higher ethanolic solution concentrations could decrease anthocyanin extraction. Similar behavior was found by Khazaei et al. [33]. The improvement of anthocyanin extraction with increasing ethanol concentration can be related to the increase in molecular polarity and solubility of the anthocyanins in extraction solution. However, the use of 90% ethanolic solution promoted lower anthocyanin extraction that could be explained by the decrease in solution polarity and, consequently, anthocyanin solubility [33].

Thus, for agitated bed extraction, the independent variable which more influenced anthocyanin content was ethanol concentration, followed by solid:liquid ratio.

According to Figure 1, the increase in solid:liquid ratio also promoted the increase in total monomeric anthocyanins. When the volume of extraction solution increases, the mass transfer increases as a result of higher concentration gradient, consequently increasing the diffusion rate, which is directly proportional to this gradient [34].

Moreover, temperature also showed a slight positive effect on anthocyanin extraction, with higher values for extractions performed above 40 °C, since the increase in temperature promotes the increase in the cell membrane permeability, making the mass transfer easier.

According to ANOVA, the results of antioxidant capacity measured by both methods could not obtain predictive models (R^2^ = 0.617; 0.362 for the Folin–Ciocalteu and ABTS+ assay, respectively). Therefore, it was not possible to have response surfaces.

**Figure 2:**
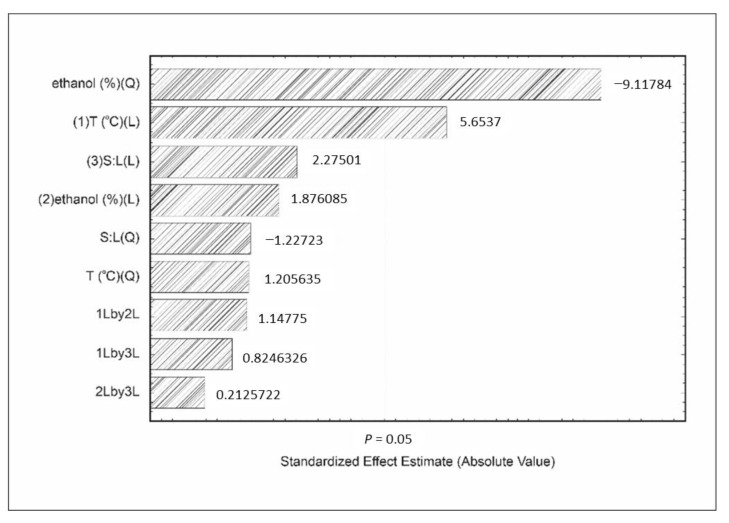
Effect of independent variables on Folin–Ciocalteu reducing capacity assay, where temperature (T) and solid:liquid ratio (S:L).

**Figure 3:**
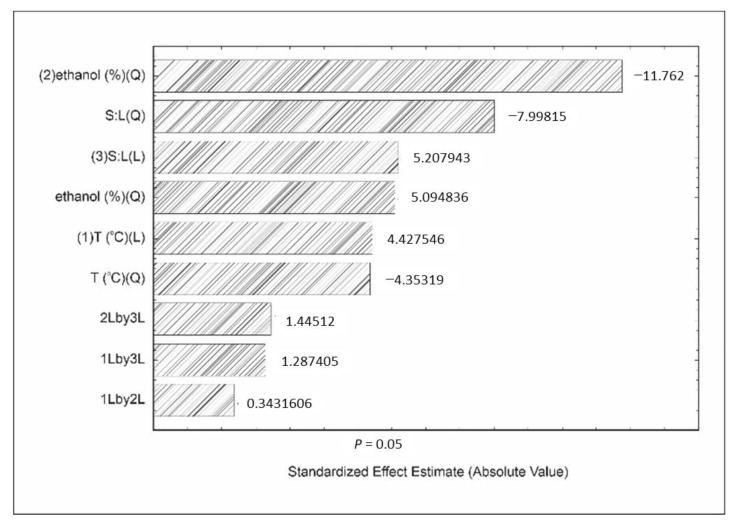
Effect of independent variables on ABTS+ cationic radical scavenging activity, where temperature (T) and solid:liquid ratio (S:L).

The effect of variables affecting these responses was illustrated by Pareto charts (Figure 2 and Figure 3), which are generally used to indicate the most relevant variables affecting the processes [35], besides estimating the impact of each corresponding factor [36,37].

Folin–Ciocalteu reducing capacity results varied from 2865 to 10,565.22 mg gallic acid 100 g^−1^ (Table 1). Lenquiste et al. [38] analyzed two different types of jaboticaba extract and observed values in the same range (4861 mg gallic acid equivalent 100 g^−1^ for methanolic extract and 3612 mg gallic acid equivalent 100 g^−1^ for aqueous extract).

Ethanol concentration and temperature affected the Folin–Ciocalteu reducing capacity (Figure 2). Similarly to anthocyanins, this response was linearly affected by the increase in temperature. Higher temperatures led to better extraction yields due to the increase in cell membrane permeability, as well as the increase in solubility and the diffusion rate [31,39,40,41].

The quadratic ethanol concentration showed a negative effect on the Folin–Ciocalteu reducing capacity, indicating that the increase of this variable would lead to higher results, up to a maximum value, above which this response would show an opposite behavior, as also observed for anthocyanins.

The values observed for ABTS+ antioxidant capacity varied from 184.68 to 1561.59 μmol Trolox equivalent g^−1^, which are in the same range than those described by Inada et al. [3] after studying all fractions of jaboticaba, including jaboticaba peel (976 μmol of Trolox equivalent g^−1^ of peel dry weight). However, these values are lower than described by Leite-Legatti et al. [42] (9458 μmol Trolox equivalent g^−1^ of peel dry weight).

Antioxidant capacity measured by ABTS+ assay was significantly affected by the solid:liquid ratio, ethanol concentration, and temperature (*p* < 0.05). According to Figure 3, ethanol concentration was the variable that most influenced this response. On the other hand, the increase in ethanol concentration was responsible for decreasing the antioxidant capacity by ABTS+. Ethanol increasing up to 60% highly promoted the increase in polar difference between some compounds and ethanolic solution, resulting in the decrease in the diffusion coefficient and compound solubility. These compounds are responsible for antioxidant activities. This phenomenon could be explained as a consequence of the lower solubility of compounds in ethanol than in water [43].

Considering that our main purpose was to obtain an anthocyanin-rich extract, this response was considered for choosing the best process conditions. Thus, based in our previous discussion (Figure 1a,c), the condition selected as the most adequate for anthocyanin extraction from jaboticaba skin was: temperature of 50 °C, 74% ethanol, and a solid:liquid ratio of 1:13.

### 3.2. Non-Conventional Extraction

#### 3.2.1. UAE

**Figure 4:**
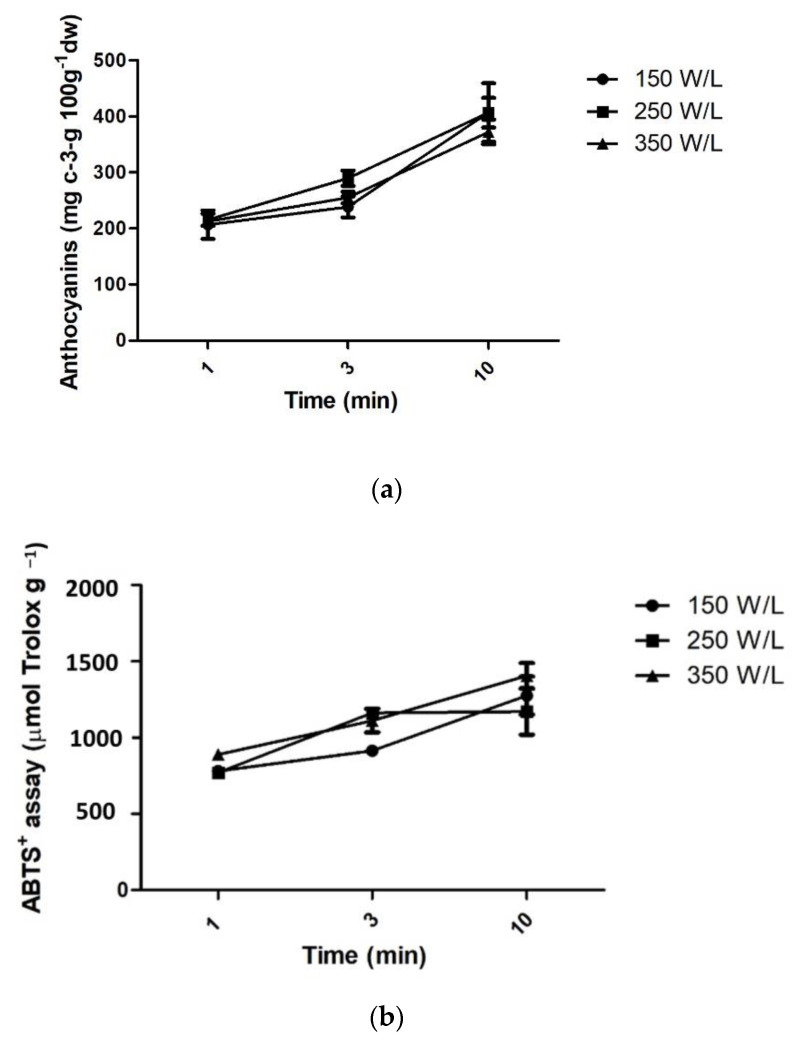

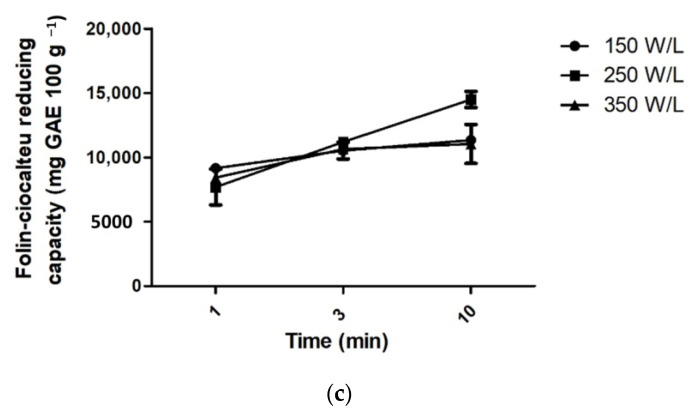
Total monomeric anthocyanins content (**a**), ABTS+ (**b**), and Folin–Ciocalteu reducing capacity (**c**) of jaboticaba skin extracted by UAE.

Figure 4 shows the results for UAE. Total monomeric anthocyanins in the extracts varied from 14.24 to 407.15 mg of cyanidin-3-glucoside 100 g^−1^, Folin–Ciocalteu reducing capacity varied from 3618.14 to 14,496.47 mg GAE 100 g^−1^ and antioxidant capacity by ABTS+ assay varied from 288.68 to 1406.20 μmol Trolox g^−1^. According to Figure 4, the increase in ultrasound power did not affect total monomeric anthocyanins content being not statistically significant (*p* < 0.05), while the increase in processing time resulted in better yields.

Acoustic cavitation is the phenomena responsible for the improvement on the extraction yield in the ultrasound-assisted extraction. The propagation of ultrasound waves through a liquid generates consecutive cycles of compression and rarefaction, which form cavitation bubbles. These bubbles keep on growing in subsequent cycles, resulting in a violent collapse at very high pressures (50–1000 atm) and temperatures (around 5000 K), which generates high-speed liquid jets, promoting shockwave damage and structural changes in the solid surface [43]. According to Chemat et al. [44], the different cavitation-induced mechanisms responsible for the improvement on the extraction efficiency are fragmentation, detexturation, erosion, capillarity, and sonoporation, which can occur independently or simultaneously, considerably increasing the mass transfer.

Ultrasound power has been reported as one of the most important process variables affecting UAE. In general, the yield of UAE extraction increases with the increase in power up to a peak value, above which it eventually decreases or reaches a plateau [45,46]. The increase in power leads to a higher acoustic cavitation effect, which produces more violent breakdown and, consequently, higher extraction yield. González-Centeno et al. [13], for example, reported a linear effect of power on the extraction yield of phenolic, total flavonoids, and total antioxidants from grape pomace. Zou et al. observed a linear increase in the melanin yield extracted from *Auricularia auricula* by UAE, with the increase in power [47]. However, power intensity above the peak value may decrease or have no effect on the extraction yield, since the excessive number of cavitation bubbles can increase the inter-bubble collision, reducing the impacts of bubble implosion. In addition, these bubbles can surround the probe and hinder energy transmission [48].

In addition, high temperatures and pressures can also promote phenolic oxidation, resulting in the formation of free radicals which can attack the target compounds, resulting in their degradation [32,49]. Higher temperatures can also be responsible for the formation of solvent vapors that enter the cavitation bubbles, reducing the pressure gradient between the inside and outside of the bubbles. The decrease in surface tension and the increase in vapor pressure reduces the sonochemical effects, producing less cavitational energy conversion [45,46]. This can also explain the results of the present work, which showed no effect of the power density on the extraction of antioxidant compounds. These results are similar to those observed by Chen et al. [50] and Sabino et al. [49] in the UAE of anthocyanins from *Rubia sylvatica* fruits and jambolan extracts, respectively.

According to Fick’s second law, the analyte concentration is proportional to the extraction time [51], which corroborates the higher values observed for 10 min of extraction, for all the responses. However, similarly to the discussed for ultrasound power, an excessive increase in the extraction time above an optimum value could also negatively affect the extraction yield [46], mainly due to the temperature increase and the reduction in the cavitational effects, as previously explained. In the present work, the maximum extraction time studied (10 min) was not enough to promote the reduction in the recovery of antioxidant compounds.

Considering energetic costs and the absence of statistical significance among power densities, the UAE at 150 W/L could be considered the most appropriate condition. The use of lower power density reduces production costs, making it an excellent alternative for industry. Thus, considering that the highest anthocyanin values were achieved in the highest time studied in this work (10 min) and in the lowest powder density (150 W/L), these conditions were considered as the most adequate for the UAE of anthocyanins from jaboticaba skin.

#### 3.2.2. HHE

Total monomeric anthocyanins in the extracts obtained by HHE varied from 113.97 to 186.53 mg of cyanidin-3-glucoside 100 g^−1^, Folin–Ciocalteu reducing capacity varied from 5736.90 to 9176.30 mg GAE 100 g^−1^ and antioxidant capacity by ABTS method varied from 451.56 to 628.50 μmol de Trolox g^−1^.

**Figure 5:**
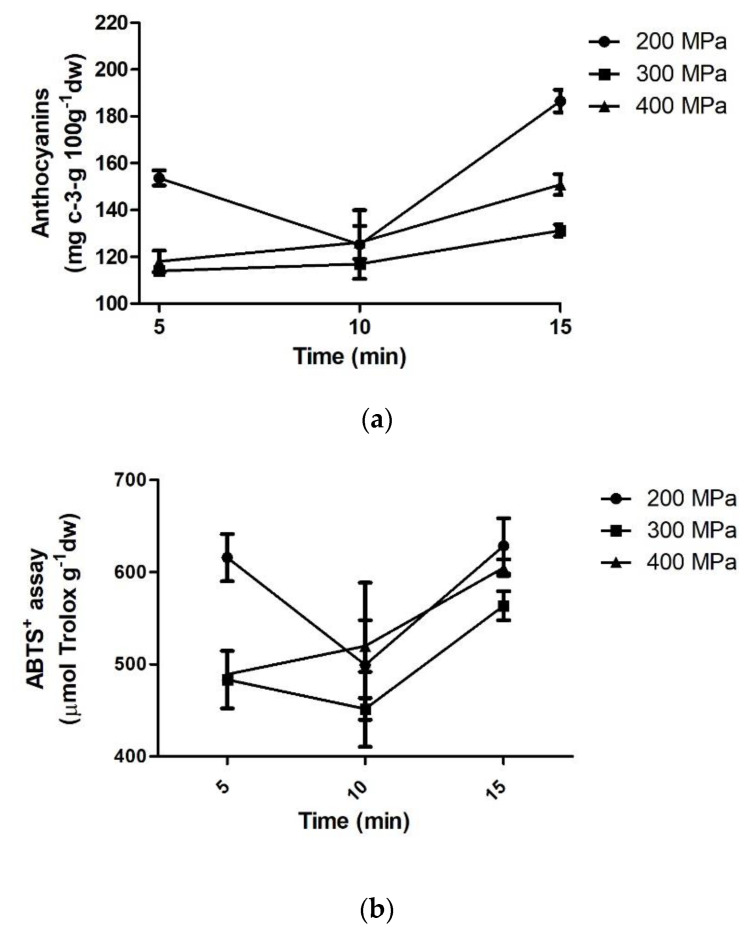

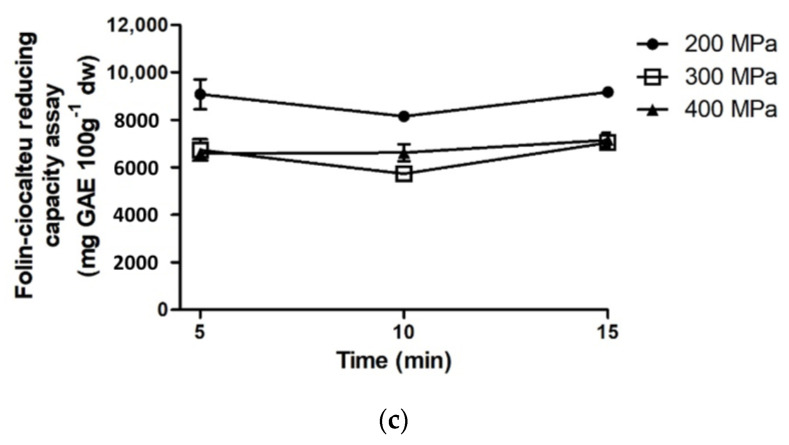
Total monomeric anthocyanins content (**a**), ABTS+ (**b**), and Folin–Ciocalteu reducing capacity (**c**) from jaboticaba skin extracted by HHE.

According to Figure 5, the highest antioxidant capacity and total monomeric anthocyanins were found for the highest extraction time. However, there was no significant difference (*p* < 0.05) in the extraction efficiency among different pressures. This can indicate that pressure increase may negatively affect extraction, thus suggesting a possible anthocyanin degradation when higher pressures are used [46]. The anthocyanin degradation is believed to be caused by its association with flavanol, forming a pyran ring. It is also suggested that pelargonidin-3-O-glucoside reacts with proteins and phenolic acids, decreasing the anthocyanins content [47,52]. Additionally, the increase in pressure promotes increase in the material temperature by an adiabatic heating effect that could explain lower anthocyanin concentrations with higher pressures [53].

However, similarly to UAE, the increase in pressure may negatively affect anthocyanin content. According to Patras et al. [53], high pressure application could be responsible for phenolic compound degradation in some fruits. This effect is a consequence of peroxidase and polyphenol oxidase activity that oxidize phenolic quinones [54]. de Jesus et al. [55] evaluated the effect of three different pressures (400, 500, and 600 MPa) on microbial inactivation and anthocyanin extraction from açaí pulp, and observed that high hydrostatic pressure was not able to completely inactivate peroxidase and polyphenol oxidase from açaí pulp.

Therefore, the yield of anthocyanin extraction by high hydrostatic pressure can be affected by several factors, such as pressure, composition of product, the action of oxidative enzymes, and time [56]. Anthocyanin extraction was positively influenced by HHE time. The increase in application time (5 min to 15 min) increased analyte extraction due to equilibrium of pressure between the inside and outside of the material or the higher diffusion speed of the solvent when shorter times were applied on HHE [54]. This trend was observed by Briones-Labarca et al. [57] that studied HHE of Chilean papaya seeds using 500 MPa and different extraction times (5, 10, and 15 min). They reported that the extraction yields were higher when the time of extraction increased from 5 to 15 min.

### 3.3. Comparison of Conventional and Non-Conventional Methods

According to Table 3, ultrasound-assisted was the best extraction technology for anthocyanins recovery and a satisfactory antioxidant capacity value. The results suggested that UAE is a time-saving extraction technology, besides being a more efficient extraction when compared with agitated-bed and high hydrostatic pressure-assisted extraction.

However, this technology is still not widely implemented in the food industry. Some studies suggested that scaling-up the process is a promising alternative to increase industry confidence [58]. Plazzotta and Manzocco [59] reported that, despite the increased initial investment, the energy costs are lower than for the conventional extraction methods. Thus, this technology can have a good cost benefit and deserves to be further investigated.

### 3.4. Identification of the Anthocyanins

**Figure 6:**
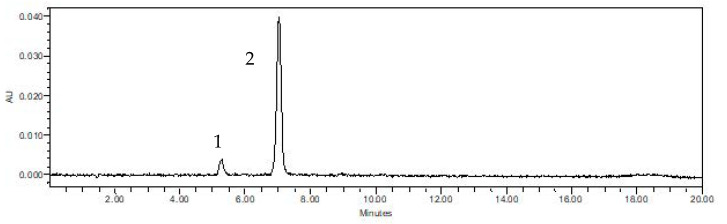
Monomeric anthocyanin chromatogram of jaboticaba extract by UAE, at 520 nm, where **peak 1** is delphinidin-3-O-glucoside and **peak 2** is cyanidin-3-O-glucoside.

After ultrasound-assisted technology has been chosen as the best extraction technology among analyzed methos, two anthocyanins were identified (Figure 6).

According to the chromatogram, it was possible to identify by comparison of retention times, making it possible to identify delphinidin-3-O-glucoside (14 mg 100 g^−1^ dw–**Peak 1**) and cyanidin-3-O-glucoside (393 mg 100 g^−1^ dw–**Peak 2**). Cyanidin-3-O-glucoside was the major anthocyanin as reported by Inada et al. [3] and Paludo et al. [60]. This result corroborates the findings of Paludo et al. [60] and Albuquerque et al. [30]. Likewise, cyanidin-3-O-glucoside was the majority anthocyanin found on UAE (97%), delphinidin-3-O-glucoside was the second (3%).

## 4. Conclusions

UAE and HHE promoted higher monomeric anthocyanin extraction in shorter times compared to agitated bed extraction, making them potential alternatives to improve bioactive compound extraction and, consequently, antioxidant capacity. UAE showed the best results, mainly being affected by the extraction time, while the ultrasound intensity did not significantly affect the extraction yield, suggesting that the lowest power setting was enough to improve the performance of the extraction process. However, further studies are necessary to evaluate the economic viability and upscaling of these processes.

## Figures and Tables

**Table 1:** Results found from 17 tests using agitated bed extraction.

	Independent Variables			Responses		
Tests	Temperature (°C)	Ethanol Concentration (%)	Solid: Liquid Ratio	Anthocyanins (mg c-3-g 100 g^−1^ dw)	Folin–Ciocalteu Reducing Capacity (mg GAE 100 g^−1^ dw)	ABTS^+^ Assay (μmol Trolox g^−1^ dw)
1	30	26	1:7	87 ± 3	3100 ± 13	270 ± 9
2	50	26	1:7	113 ± 5	3500 ± 74	280 ± 12
3	30	74	1:7	232 ± 6	4400 ± 62	339 ± 17
4	50	74	1:7	249 ± 8	5900 ± 153	374 ± 29
5	30	26	1:13	125 ± 3	3400 ± 112	238 ± 17
6	50	26	1:13	143 ± 3	4700 ± 398	329 ± 23
7	30	74	1:13	246 ± 7	5100 ± 324	397 ± 31
8	50	74	1:13	284 ± 5	7000 ± 209	502 ± 25
9	23	50	1:10	192 ± 1	7000 ± 135	407 ± 7
10	57	50	1:10	225 ± 9	10,600 ± 474	666 ± 50
11	40	10	1:10	59 ± 1	5300 ± 96	1600 ± 143
12	40	90	1:10	192 ± 2	2900 ± 93	185 ± 16
13	40	50	1:5	169 ± 3	7300 ± 279	238 ± 2
14	40	50	1:15	258 ± 4	8100 ± 218	595 ± 45
15	40	50	1:10	223 ± 8	6900 ± 315	563 ± 38
16	40	50	1:10	228 ± 7	7400 ± 222	518 ± 14
17	40	50	1:10	224 ± 7	6300 ± 27	480 ± 19

**Table 2:** Coefficient second-order regression for total anthocyanins monomeric content, Folin–Ciocalteu reducing capacity and ABTS+ assay.

Coefficient	Anthocyanins (mg c3g 100 g^−1^ dw)	Folin–Ciocalteu Reducing Capacity (mg GAE 100 g^−1^)	ABTS^+^ Assay (μmol Trolox g^−1^)
β_0_	224.41	7005.03	539.04
β_1_	11.26	830.08	49.57
β_2_	56.19	277.72	−132.77
β_3_	19.53	336.77	58.79
β_11_	−4.58	N.S	−52.86
β_22_	−34.08	−1501.22	63.97
β_33_	N.S	N.S	100.42
β_12_	N.S	N.S	N.S
β_13_	N.S	N.S	N.S
β_23_	N.S	N.S	N.S
R^2^ predicted	0.9490	0.6170	0.3620
R^2^ adjusted	0.8880	0.1419	0
Lack of fit	109.29	16.76	116.60

N.S: Non-significant (*p* > 0.05).

**Table 3:** Anthocyanins, Folin–Ciocalteu reducing capacity, ABTS+ from extracts obtained by conventional and non-conventional methods.

Method	Condition	Anthcyanins ^1^	Folin–Ciocalteu ^2^	ABTS^+ 3^
Conventional	1 h	284 ± 5 ^c^	7000 ± 209 ^c^	502 ± 25 ^b^
High Hydrostatic Pressure	200 MPa 15 min	187 ± 8 ^b^	9200 ± 481 ^b^	628 ± 25 ^b^
Ultrasound	150 W/L 10 min	407 ± 91 ^a^	11,300 ± 428 ^a^	1300 ± 216 ^a^

^1^ The anthocyanin content was expressed as mg of cyanidin-3-O-glucoside equivalent per 100 g of dry weight (mg c3g. 100 g^−1^ dw); ^2^ Folin–Ciocalteu reducing capacity was expressed as mg of gallic acid equivalent per 100 g of dry weight (mg GAE 100 g^−1^ dw); ^3^ ABTS+ cationic radical scavenging activity was expressed as μmol Trolox equivalent per grams of dry weight (μmol Trolox g^-^). Different letter indicates significant difference between extracts obtained by different methods (*p* ≤ 0.05).

## Data Availability

All the data can be found in this study. Any inquiries or additional data can be requested from the corresponding author.
